# Diaphragmatic Hernia after Laparoscopic Esophagomyotomy for Esophageal Achalasia in Pregnancy

**DOI:** 10.5402/2011/871958

**Published:** 2010-10-18

**Authors:** Meena Khandelwal, Chad Krueger

**Affiliations:** Department of Obstetrics & Gynecology, Cooper University Hospital, University of Medicine & Dentistry of New Jersey, 1 Cooper Plaza, 623 Dorrance Building, Camden, NJ 08103, USA

## Abstract

*Background*. The optimal treatment for management of esophageal achalasia in pregnancy is controversial. Little information exists about pregnancy outcome after successful myotomy. *Case*. Achalasia in pregnancy was diagnosed when a patient presented with pneumomediastinum from microrupture of the overdistended esophagus. An attempt at surgical correction failed due to the development of aspiration pneumonia with general anesthesia. Conservative medical therapy was undertaken, but fetal growth restriction developed. The patient underwent interval surgical correction, but subsequent pregnancy 6 months later was complicated by acute diaphragmatic hernia necessitating preterm delivery. *Conclusion*. Prior to surgery in pregnancy, emptying the dilated esophagus via nasoesophageal tube suctioning maybe warranted to avoid aspiration. Women, despite having undergone successful myotomy, should be counseled on the risks of pregnancy and to avoid pregnancy for at least 1 year thereafter.

## 1. Introduction

Diaphragmatic hernia is fortunately a rare but devastating occurrence in pregnancy. The causes in reported cases result from trauma, congenital, or hiatal (paraesophageal) defect [1]. We report the first case of diaphragmatic hernia in pregnancy after surgical correction for esophageal achalasia. Achalasia is a rare disorder of the esophagus and lower esophageal sphincter that affects between 0.03 and 1/100,000 persons per year [2]. While the exact pathophysiology is unknown, the underlying mechanism involves the destruction of the myenteric plexus leading to the absence of esophageal peristalsis and the failure of the lower esophageal sphincter (LES) to relax resulting in possible increased pressure at the LES [2]. The dysphagia, chest pain, regurgitation, nausea, and vomiting associated with this disease lead to malnutrition which, during pregnancy, can lead to increased morbidity and mortality for both the mother and the fetus. We report on two pregnancies in a case of achalasia diagnosed during pregnancy. This case highlights the different treatment modalities that can be used for achalasia, tailored to gestational age at diagnosis, and reflects on the risks and complications in current and future pregnancies.

## 2. Case Report

**Figure 1:**
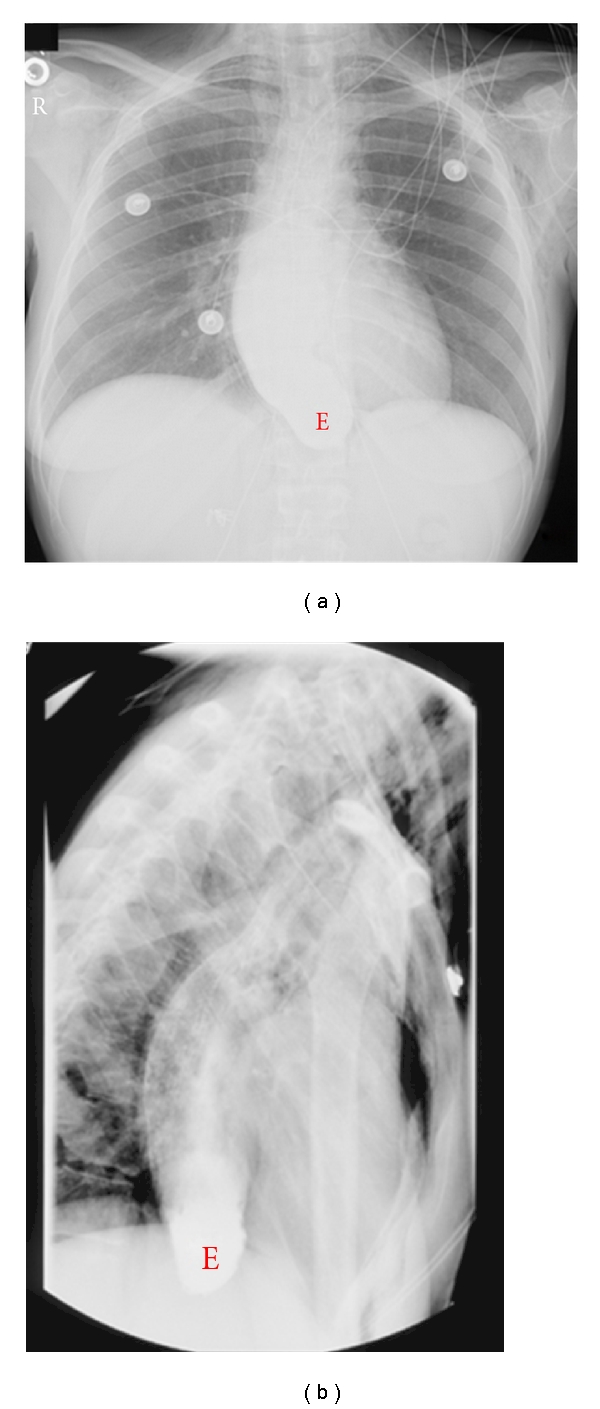
(a) An esophagogram showing the dilated esophagus (labeled “E”). The distal esophagus forms a “bird's beak” at the lower esophageal sphincter and contains a column of barium that could not pass into the stomach. (b) Lateral view of dilated esophagus with barium column.

Informed consent was obtained from the patient. A 22-year-old para 2 at 15 weeks gestation with dichorionic diamniotic twins was transferred to our hospital after several days of intractable vomiting and 30 lbs weight loss over the past few months despite the pregnancy. Her past medical history was significant for two years of frequent vomiting. Chest X-ray revealed pneumomediastinum without any respiratory distress. A barium esophagogram was negative for esophageal perforation but was consistent with esophageal achalasia (Figure 1). A manometry study confirmed the diagnosis of esophageal achalasia, and esophagogastroduodenoscopy showed Candida esophagitis. This was treated with intravenous Fluconazole for 2 weeks which significantly improved her oral intake and weight, allowing for conservative management.

**Figure 2:**
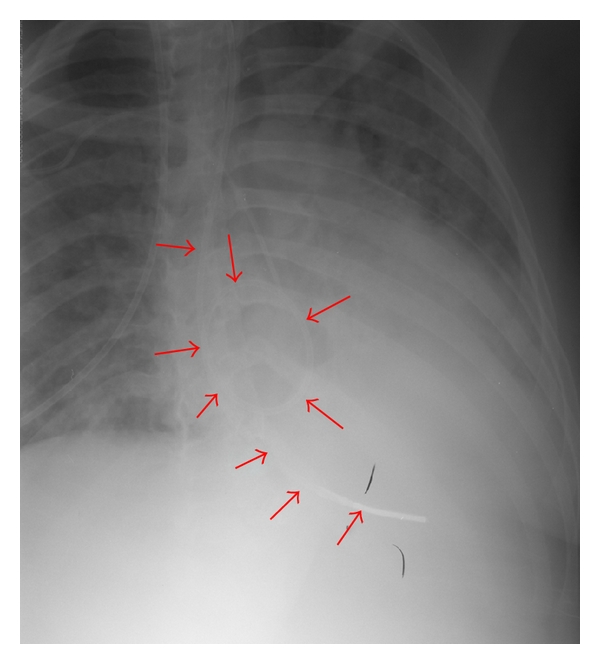
The Dophoff tube is coiled within the dilated esophagus. The tube's path is outlined with the red arrows.

**Figure 3:**
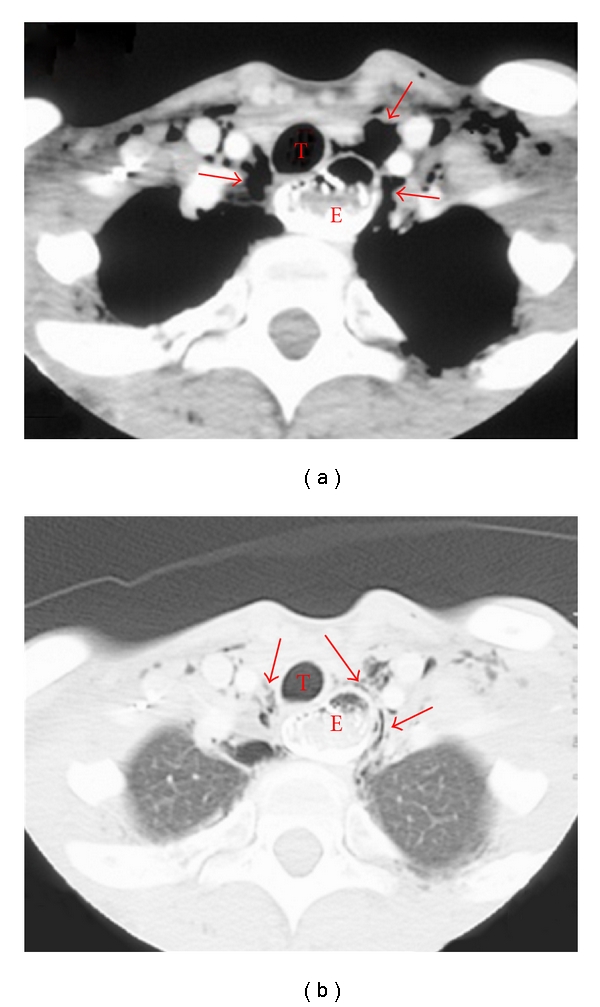
(a) A CT showing a dilated esophagus (labeled “E”) and pneumomediastinum (shown with the arrows). There is air, fluid, and solid food within the esophagus. The trachea is labeled “T” for reference. (b) Another CT scan showing the dilated esophagus and the pneumomediastinum.

She was readmitted during the 20th week with further weight loss and malnutrition. Total parenteral nutrition (TPN) was started, and laparoscopic lower esophageal myotomy was scheduled. During the attempted induction of anesthesia with cricoid pressure, the patient aspirated esophageal contents and required intensive unit care for aspiration pneumonia. Insertion of a Dophoff tube was attempted for continued gastrointestinal nutrition. Two attempts failed because the tube could not be passed through the LES but coiled in the large dilated esophagus (Figure 2). CT scan confirmed the presence of pneumomediastinum (Figure 3), and so pneumatic dilation or Botulinium injections were not considered. The patient declined the passage of the tube under endoscopic guidance, or any further surgical treatment. She was started on oral Nifedipine before meals which helped her gain weight.

Although she gained 15 lbs at home, she was readmitted during the 29th week for intrauterine growth restriction of the twins. Intravenous hyperalimentation was reinitiated, and a Cesarean section was scheduled for the 34th week of her pregnancy. On the morning of the scheduled delivery, intrauterine fetal demise of 1 twin was diagnosed. The other fetus was delivered via Cesarean section later that day. The male baby weighed 1529 grams (<10th percentile) and required basic neonatal care for 2 weeks in the hospital. Robotic thorascopic-assisted Heller esophagomyotomy with a flexible esophagoscopy was performed successfully 5 months postpartum. She did well postoperatively and gained significant weight thereafter.

**Figure 4:**
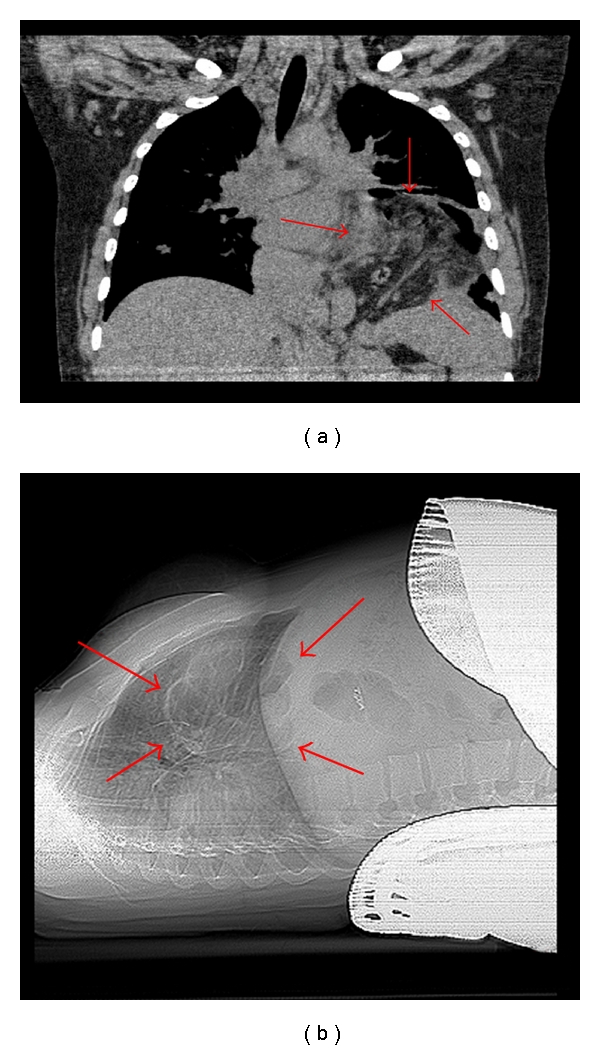
(a) CT of the chest showing the large diaphragmatic hernia (shown by the arrows). The hernia is compressing the left lung and has caused some slight deviation of the trachea to the contralateral side of the chest. (b) Lateral view of diaphragmatic hernia (outlined by arrows) with patient laying supine.

Six months later she conceived again and presented at 25 weeks gestation with recurrent intractable vomiting for 2 weeks and 12 lbs weight loss. Esophagogastroduodenoscopy revealed a duodenal ulcer and small hiatal hernia. The hernia was suspected to be a complication of the myotomy surgery <1 year ago. Conservative management including proton-pump inhibitors was initiated. Ten days later, the patient started to experience severe LUQ, left shoulder pain, and mild respiratory distress. A chest CT revealed a large hiatal diaphragmatic hernia with loops of bowel that were compressing the base of the left lung (Figure 4). Nasogastric tube was inserted to decompress the bowel and possibly decrease her respiratory symptoms, allowing her pregnancy to advance further. Surgeons did not feel the diaphragmatic repair could safely be performed in the gravid state due to the large amount of intestines herniated into the chest. Due to worsening respiratory symptoms and compression atelectasis over the next 10 days, she was given betamethasone for fetal lung maturity, and a repeat Cesarean section with bilateral tubal ligation was performed at 28-week gestation. A male baby weighing 1372 grams (24th percentile) was delivered. Two days postpartum, the mother underwent uneventful repair for the diaphragmatic hernia. The infant required about 8 weeks in neonatal intensive unit care and developed mild bronchopulmonary dysplasia and patent ductus arteriosus that was later ligated. The mother and the baby were doing well 4 years later.

## 3. Discussion

Achalasia is an esophageal motility disorder most commonly presenting with dysphagia for liquids and solids, chest pain and regurgitation. Patients might also state that it takes them much longer to finish meals than everyone else or that they need to stretch, stand up, or move around immediately after eating [2]. Diagnosis is usually made based on the patient's symptoms, but since these symptoms mimic those of more common diseases, diagnosis may be delayed for up to 2-3 years. Pregnancy can make the diagnosis of achalasia more difficult as the increased levels of progesterone relax the LES, decreasing its resting tone and alleviating some of the symptomatology [3]. This could explain why our patient did not complain of dysphagia. However, she reported eating frequently but had postprandial vomiting of large amounts of undigested food. She was hungry all the time as very little food actually passed the LES into the stomach and beyond for absorption. This resulted in weight loss and malnutrition. The symptoms of achalasia can also be falsely attributed to hyperemesis gravidum as reported by Ohno and colleagues [4]. 

A barium study shows the distal esophagus tapering into a “bird's peak,” a column of contrast sitting in the esophageal lumen, and a variable amount of proximal esophagus dilation (Figure 1). Manometry is considered to be the “gold standard” for achalasia diagnosis and will show an absence of esophageal peristalsis and incomplete LES relaxation [5].

 Since we are currently unable to restore the myenteric plexus for disease cure, the goal of any achalasia treatment is to facilitate the passage of food through the esophagus and into the stomach. Pharmacologic agents such as long-acting nitrates, calcium channel blockers (nifedipine used in our case), anticholinergics, beta-adrenergic agonists, theophylline, and sildenafil, a phosphodiesterase inhibitor, have limited short-term clinical value in the treatment of achalasia but have poor long-term efficacy and significant side effects. They are currently only recommended for use in patients who are not able or willing to undergo a more definitive therapy [2, 5]. Botulinum toxin injections have a much greater success rate than pharmacologic treatment and a lower morbidity and mortality than pneumatic dilation and surgical myotomy [2]. However, over 50% of patients injected with the toxin develop recurrent symptoms within 6 months, and the injection can make further surgical treatments of the esophagus more difficult to perform [5]. In addition, effects of the Botulinum toxin on the fetus are unknown, making the treatment less attractive in pregnancy though its use in pregnancy has been reported [6]. 

Forceful dilatation is the oldest form of surgical treatment for achalasia. Such treatment is now performed via pneumatic balloon dilatation and is usually an outpatient procedure [2]. While the initial success rate is comparable to lower esophageal myotomy, most studies show that myotomy has a greater long-term success rate [2]. With a history of pneumomediastinum in our patient, there was a concern about esophageal perforation, a known complication of pneumatic balloon dilatation [3]. Surgical lower esophageal myotomy relieves achalasia by making a longitudinal transection of the lower esophageal sphincter [5]. Most surgeons will add a Toupet or Dor fundoplication to the myotomy, though not necessary, in an attempt to decrease acid reflux which is a possible side effect of the procedure [2, 5, 7]. Since this procedure can be performed laparoscopically, with a high success rate and low mortality, it has become the treatment of choice for achalasia [2]. 

Achalasia in pregnancy has been described as presenting either in the 1st or the 3rd trimester with outcome being favorable in the latter [8]. Satin et al. reported on a patient who presented with symptoms of achalasia at 38-week gestation. Labor was induced and healthy baby was delivered. She was treated by pneumatic dilation postpartum [3]. However, Ohno and coworkers reported intrauterine fetal demise at 27 weeks, despite treatment with intravenous hyperalimentation [4]. She subsequently underwent a successful myotomy-fundoplasty and had an uneventful pregnancy a year later. Delaying definitive treatment for achalasia until after the delivery of the baby seems to be an appropriate strategy when the symptoms first present during the third trimester. However, when the symptoms first appear during the 1st or 2nd trimester, it seems that a definitive treatment should be sought to decrease the morbidity and mortality to the fetus. Only 1 prior report of Candida esophagitis as a complication of achalasia has been reported [9]. Treatment results in symptomatic improvement—for 7-8 weeks and a healthy infant outcome at term in Kalish's case but only 4 weeks in our case. 

Successful treatment with pneumatic dilation during pregnancy with delivery of healthy term babies has been described [10–13]. No other form of treatment during pregnancy has been described. It seems logical that, as the procedure gains in popularity and surgeon experience, the laparoscopic lower esophageal myotomy could also be used in similar situations. However, surgical treatment requires anesthesia with its associated risk of aspiration. The megaesophagus, with its undigested contents under pressure, adds significantly to the pregnancy-associated risk of aspiration as in our case. Overnight decompression of the esophagus, with nasoesophageal tube, may reduce intraesophageal pressure and related risk of aspiration. 

Our patient's pregnancy after her esophageal myotomy is also worthy of discussion. Diaphragmatic hernia after myotomy surgery has been reported acutely and as a late complication in the nonpregnant state [14, 15]. The paraesophageal diaphragmatic hernia most likely resulted from a weakness in the diaphragm from the myotomy surgery [16, 17]. Widening of the esophageal hiatus in the diaphragm could have resulted/worsened from the short interval of myotomy-to-pregnancy, increased abdominal pressure from the enlarging uterus, hyperemesis, and increased progesterone levels. How pregnancy might affect the healing of a myotomy scar or how long to wait after, before it is safe to get pregnant, is unknown. 

Acute diaphragmatic hernia occurring during pregnancy is associated with high morbidity and mortality for the mother and fetus [18, 19]. In a review of cases of diaphragmatic hernia occurring during pregnancy, Eglinton and colleagues reported that 67% cases occur in multiparous women, left-sided were more common (94%), and the most common presentation is unremitting vomiting [1]. There is large variability in treatment strategies. In the cases reviewed, diaphragmatic hernia was repaired antepartum in 20%, postpartum in 50%, and 20% received nonoperative treatment. The treatment decision was primarily based on the symptoms of the mother and the period of gestation. A patient presenting with acute dyspnea due to compression atelectasis, mediastinal shift causing a decrease in cardiorespiratory function that is not relieved by decompression via nasogastric suction, or visceral strangulation, should undergo emergency surgery regardless of gestational age [1]. A patient with emergency presentation but without evidence of strangulation maybe managed conservatively with nasogastric suction in a tertiary care center under close supervision for the sole purpose of achieving fetal lung maturity. We were able to manage our acute patient conservatively for 3 weeks, after which she started desaturating despite all efforts. 

 For asymptomatic patients with diaphragmatic hernia which usually is the chronic form, management is more controversial. It has been recommended by some that they undergo a Cesarean section once fetal lung maturity has been achieved [18]. This approach was based on the premise that labor causes an increase in intra-abdominal pressure, leading to further herniation and an increased risk of death as reported in some cases. However, as Eglinton and colleagues suggest, it is unknown how many women with undiagnosed diaphragmatic hernias labor successfully, making it difficult to prove that the risk of surgery to repair the hernia is less than the risk of leaving the hernia alone [1]. 

In summary, our case illustrates the significant morbidity associated with achalasia in pregnancy. This is also the first reported case of diaphragmatic hernia in subsequent pregnancy as a result of surgical management of achalasia.

##  Conflict of Interests

The authors declare that there is no conflict of interests.
